# Omega-3 fatty acids and major depression: A primer for the mental health professional

**DOI:** 10.1186/1476-511X-3-25

**Published:** 2004-11-09

**Authors:** Alan C Logan

**Affiliations:** 1 Integrative Care Centre of Toronto, 3600 Ellesmere Road, Unit 4, Toronto, ON M1C 4Y8, Canada

## Abstract

Omega-3 fatty acids play a critical role in the development and function of the central nervous system. Emerging research is establishing an association between omega-3 fatty acids (alpha-linolenic, eicosapentaenoic, docosahexaenoic) and major depressive disorder. Evidence from epidemiological, laboratory and clinical studies suggest that dietary lipids and other associated nutritional factors may influence vulnerability and outcome in depressive disorders. Research in this area is growing at a rapid pace. The goal of this report is to integrate various branches of research in order to update mental health professionals.

## Introduction

Major depressive disorder (MDD) is a recurrent, debilitating, and potentially life threatening illness. Over the last 100 years, the age of onset of major depression has decreased, and its overall incidence has increased in Western countries. The increases in depression, up to 20-fold higher post 1945, cannot be fully explained by changes in attitudes of health professionals or society, diagnostic criteria, reporting bias, institutional or other artifacts [1,2] Despite advances in pharmacotherapy, and the increasing sophistication of cognitive/behavioral interventions, there are many patients with MDD who remain treatment resistant [3].

Depression is undoubtedly an extremely complex and heterogeneous condition. This is reflected by the non-universal results obtained using cognitive-behavior and antidepressant medications. As research continues to mount, it is becoming clear that neurobiology/physiology, genetics, life stressors, and environmental factors can all contribute to vulnerability to depression. While much attention has been given to genetics and life stressors, only a small group of international researchers have focused on nutritional influences on depressive symptoms. Collectively, the results of this relatively small body of research indicate that nutritional influences on MDD are currently underestimated [4]. Omega-3 fatty acids in particular represent an exciting area of research, with eicosapentaenoic acid (EPA) emerging as a new potential agent in the treatment of depression [5].

## Omega-3 fatty acids

Omega-3 fatty acids are long-chain, polyunsaturated fatty acids (PUFA) of plant and marine origin. Because these essential fatty acids cannot be synthesized by the human body, they must be derived from dietary sources. Flaxseed, hemp, canola and walnut oils are all generally rich sources of the parent omega-3, alpha linolenic acid (ALA). Dietary ALA can be metabolized in the liver to the longer-chain omega-3 eicosapentaenoic (EPA) and docosahexaenoic acid (DHA). This conversion is limited in human beings, it is estimated that only 5–15% of ALA is ultimately converted to DHA [6]. Aging, illness and stress, as well as excessive amounts of omega-6 rich oils (corn, safflower, sunflower, cottonseed) can all compromise conversion [7]. Dietary fish and seafood provide varying amounts of pre-formed EPA and DHA as highlighted in Table 1.

**Table 1:** Various Sources of EPA and DHA

Fish/Seafood	Total EPA/DHA (mg/100 g)
Mackerel	2300
Chinook salmon	1900
Herring	1700
Anchovy	1400
Sardine	1400
Coho salmon	1200
Trout	600
Spiny lobster	500
Halibut	400
Shrimp	300
Catfish	300
Sole	200
Cod	200

USDA Nutrient Database http://www.nal.usda.gov/fnic/foodcomp/search/

The dietary intake of omega-3 fatty acids has dramatically declined in Western countries over the last century, the North American diet currently has omega-6 fats outnumbering omega-3 by a ratio of up to 20:1. There are a number of reasons for this skewed ratio, most notably the mass introduction of the aforementioned omega-6 rich oils into the food supply, either directly or through animal rearing practices [8]. The ideal dietary ratio of omega-6 to omega-3 has been recommended by an international panel of lipid experts to be approximately 2:1 [9]. Given that approximately 20% of the dry weight of the brain is made up of PUFA and that one out of every three fatty acids in the central nervous system (CNS) are PUFA, the importance of these fats cannot be argued [7]. Considering that highly-consumed vegetable oils have significant omega-6 to omega-3 ratios (see Table 2), it is quite plausible that, for some individuals, inadequate intake of omega-3 fatty acids may have neuropsychiatric consequences. While far from robust at this time, emerging research suggests that omega-3 fatty acids may be of therapeutic value in the treatment of depression.

**Table 2:** Omega-6 and Omega-3 Content (%) of Dietary Oils

Oil	Omega-6	Omega-3
Safflower	75	0
Sunflower	65	0
Corn	54	0
Cottonseed	50	0
Sesame	42	0
Peanut	32	0
Soybean	51	7
Canola	20	9
Walnut	52	10
Flax	14	57

USDA Nutrient Database http://www.nal.usda.gov/fnic/foodcomp/search/

## Epidemiological Data

A number of epidemiological studies support a connection between dietary fish/seafood consumption and a lower prevalence of depression. Significant negative correlations have been reported between worldwide fish consumption and rates of depression [10]. Examination of fish/seafood consumption throughout nations has also been correlated with protection against post-partum depression [11], bipolar disorder [12] and seasonal affective disorder [13]. Separate research involving a random sample within a nation confirms the global findings, as frequent fish consumption in the general population is associated with a decreased risk of depression and suicidal ideation [14]. In addition, a cross-sectional study from New Zealand found that fish consumption is significantly associated with higher self-reported mental health status [15].

Not all studies support a connection between omega-3 intake and mood. A recent cross-sectional study of male smokers, using data collected between 1985 and 1988, indicated that subjects reporting anxiety or depressed mood had higher intakes of both omega-3 and omega-6 fatty acids [16]. In a large population-based study of older males aged 50–69, there was no association between dietary intake of omega-3 fatty acids or fish consumption and depressed mood, major depressive episodes, or suicide [17].

The epidemiological studies which support a connection between dietary fish and depression clearly do not prove causation. There are a number of cultural, economic and social factors which may confound the results. Most significantly, those who do consume more fish may generally have healthier lifestyle habits, including exercise and stress management. Despite the limitations, the epidemiological data certainly justify a closer examination of omega-3 fatty acids in those actually with depression.

## Omega-3 status in MDD

There are a number of methods used to determine EFA status in the human body, notably the plasma and red blood cell (RBC) phospholipids. These are a reflection of dietary fatty acid intake within the preceding few weeks. While not identical, significant correlations exist between blood and brain phospholipids. A number of studies have found decreased omega-3 content in the blood of depressed patients [18-21]. Furthermore, the EPA content in RBC phospholipids is negatively correlated with the severity of depression, and the omega-6 arachidonic acid to EPA ratio positively correlates with the clinical symptoms of depression [18].

More recently, investigators have been utilizing adipose tissue as a longer term measurement of EFA intake (1–3 years). In a study of 150 elderly males from Crete, the parent omega-3 ALA adipose tissue stores were negatively correlated with depression [22]. A separate study found a negative correlation between adipose tissue DHA and rates of depression. In this case, mildly depressed adults had 34.6 percent less DHA in adipose tissue than non-depressed subjects [23].

Relationships between omega-3 status and post-partum depression have also been investigated. In a cohort of 380 Australian women, plasma DHA was investigated at 6 months post-partum. Logistic regression analysis indicated that a 1% increase in plasma DHA was associated with a 59% reduction in the reporting of depressive symptoms [24]. It is well known that during pregnancy there is a significant transfer (up to 2.2 g/day) EFAs to the developing fetus [7]. Increased risk of post-partum depressive symptoms has recently been associated with a slower normalization of DHA levels after pregnancy [25].

Suicide attempts have also been associated with low levels of RBC EPA. In a study involving 100 suicide attempt cases in China compared to 100 hospital admission controls, there was an eightfold difference in suicide attempt risk between the lowest and highest RBC EPA level quartiles [26]. The seasonality of depression and suicide has been described by investigators, with more deaths in spring and summer vs.autumn and winter. Total serum cholesterol has been highly significantly synchronized with the annual rhythms in violent suicide deaths [27]. Recently, investigators found that EFA levels also vary by season, with peaks of EPA and DHA from August to September. The parent omega-3 and 6 levels did not have a seasonal variation, suggesting a seasonal interference with delta-5-desaturase conversion. The authors of this study suggest that the seasonal variation in EPA or DHA may, in part, explain seasonality of violent suicide occurrence [28].

The overlap between cardiovascular disease and depression has also been noted, with omega-3 status emerging as a common thread. Indeed, major depression in acute coronary syndrome patients is associated with significantly lower plasma levels of omega-3 fatty acids, particularly DHA [29]. In addition, elevated homocysteine levels, a known risk factor for cardiovascular disease, has been associated with the excess omega-6 fatty acids found in the Western diet [30]. Finally, lowered intake of the parent omega-3 ALA has been associated with depression in 771 Japanese patients with newly diagnosed lung cancer [31].

It is important to note that not every study supports an association between lowered omega-3 status and depression. Two studies have actually shown significant increases in plasma and RBC omega-3 status among depressed patients [32,33]. A recent study involving depressed adolescent patients found no significant relationship between adipose tissue EFA levels and depression [34].

## Possible mechanisms of omega-3 EFA

Detailed reviews of the possible neurobehavioral mechanisms of omega-3 fatty acids have been previously published and are beyond the scope of this review [35,36]. The influence of omega-3 fatty acids within the CNS is far from completely understood, and our current knowledge is largely based on the consequences of omega-3 deficiency within animal models. There are two major areas of omega-3 fatty acid influence worthy of further discussion. The first is the importance of omega-3 fatty acids in neuronal membranes. Omega-3 fatty acids are an essential component of CNS membrane phospholipid acyl chains and are therefore critical to the dynamic structure and function of neuronal membranes [37]. Proteins are embedded in the lipid bi-layer of the cell and the conformation or quaternary structure of these proteins is sensitive to the lipid components. The proteins in the bi-layer have critical cellular functions as they act as transporters and receptors. Omega-3 fatty acids can alter membrane fluidity by displacing cholesterol from the membrane [38]. An optimal fluidity, influenced by EFAs, is required for neurotransmitter binding and the signaling within the cell [39]. EFAs can act as sources for second messengers within and between neurons [35].

The second area where omega-3 fatty acids may exert significant influence in major depression is via cytokine modulation. A growing body of research has documented an association between depression and the production these proinflammatory immune chemicals. These cytokines, including interleukin-1 beta (IL-1β), -2 and -6, interferon-gamma, and tumor necrosis factor alpha (TNFα), can have direct and indirect effects on the CNS. Some of the documented activities of these cytokines include lowered neurotransmitter precursor availability, activation of the hypothalamic-pituitary axis, and alterations of the metabolism of neurotransmitters and neurotransmitter mRNA [40]. Researchers have found elevations of IL-1β, and TNFα are associated with the severity of depression [41]. Psychological stress can cause an elevation of these cytokines. It is worth noting that various tricyclic and selective serotonin re-uptake inhibiting antidepressants can inhibit the release of these inflammatory cytokines [40].

Omega-3 fatty acids, and EPA in particular, are well documented inhibitors of proinflammatory cytokines such as IL-1 β and TNFα. In addition, it has recently been suggested that the anti-inflammatory role of omega-3 fatty acids may influence brain derived neurotrophic factor (BDNF) in depression [36]. BDNF is a polypeptide that supports the survival and growth of neurons through development and adulthood. Serum BDNF has been found to be negatively correlated with the severity of depressive symptoms [42]. Antidepressant medications and voluntary exercise can enhance BDNF, while diets high in saturated fat and sucrose, and psychological stress inhibit BDNF production [36].

## Clinical evidence

The epidemiological and laboratory studies, along with the research which shows depressed patients appear to have lowered omega-3 status, have naturally led to clinical investigations. A number of case reports have appeared in the literature, the first of which was over 20 years ago. In this initial series of case reports, flaxseed oil (source of the parent omega-3 ALA) at various dosages, was reported to improve the symptoms of bipolar depression and agoraphobia [43]. An additional case report documented an improvement in depressive symptoms during pregnancy with the use of 4 g EPA/2 g DHA per day. Interestingly, improvements in symptoms (measured via the Hamilton Rating Scale for depression – HRDS) occurred at four weeks, and with the exception of insomnia and anxious thoughts, all symptoms resolved at six weeks [44].

Despite the interesting results, there are major scientific problems with case reports, most notably the placebo response. A recently published case report published took advantage of modern brain imaging to corroborate clinical improvements. In this case a patient with treatment resistant depression was placed on a daily dose of 4 g pure EPA, and after one month there were significant improvements, including a co-morbid social phobia. After nine months the patient was reportedly symptom free. It was found that over the course of the nine months of treatment, there was a 53 percent increase in cerebral phosphomonoesters and the ratio of cerebral phosphomonoesters to phosphodiesters increased 79 percent, indicating reduced neuronal phospholipid turnover. Utilizing MRI technology, the researchers found that the EPA treatment was associated with structural brain changes, including a reduction in lateral ventricular volume. This is likely to be a result of increased phospholipid biosynthesis and reduced phospholipid breakdown [45]. Given the recent research indicating a decrease in volume in various areas of the brain of depressed patients, this is certainly an important case study [46].

A series of case reports also suggest that 1 – 4 g of pure EPA may be helpful in anorexia nervosa, a condition with the highest risk of morbidity and mortality among psychiatric disorders [47]. In all six of the cases, EPA was reported to improve mood to varying degrees. For some, discontinuing EPA therapy resulted in deteriorations in mood and other psychiatric symptoms.

An interesting study examined fish oil vs.marine oil extracted from Antarctic krill in premenstrual syndrome. Krill is similar to fish oil, with the exception that it contains naturally-occurring phospholipids, and contains more EPA per gram than standard fish oil capsules (240 mg/g EPA in krill vs.180 mg/g in standard fish oil). In the 3-month trial, patients (n = 70) received 2 g of krill oil or 2 g fish oil daily for one month, then for eight days prior to, and two days during, menstruation for the following two months. Evaluation at 45 days and three months showed that krill oil significantly improved depressive symptoms of premenstrual syndrome. The absence of significant effects of fish oil on mood suggests that the presence of the phospholipids and/or higher amounts of EPA may be responsible for the therapeutic effect of krill oil [48].

There have been some controlled studies that have examined omega-3 fatty acids and a placebo intervention in depression. The first small clinical study (n = 30) showed that four months of treatment with 9.6 g of omega-3 fatty acids (6.2 g EPA/3.4 g DHA) was of therapeutic value in bipolar disorder. Specifically, this study showed a highly significant effect in treating depression (p < 0.001 HRSD scores) [49]. In a separate double-blind, placebo-controlled study (n = 22), the addition of 2 g of pure EPA to standard antidepressant medication enhanced the effectiveness of that medication vs.medication and placebo. This 3-week study, involving patients with treatment-resistant depression, showed that EPA had an effect on insomnia, depressed mood, and feelings of guilt and worthlessness. There were no clinically relevant side effects noticed [50].

In a small pilot study (n = 30), Harvard researchers found that just 1 g of EPA could reduce aggression (modified Overt Aggression Scale) and depressive symptom scores (Montgomery-Asberg Depression Rating Scale) among borderline personality disorder patients. The results of this 2-month, placebo-controlled study are encouraging, given the difficulty in treating borderline personality disorder. It is also of note that 90 percent of participants remained in the study and no clinically relevant side effects were noticed with EPA [51].

In a double-blind, placebo-controlled trial over two months, high dose fish oil (9.6 g/day) was added to standard antidepressant therapy in 28 patients with MDD. In this study the patients who received the omega-3 fish oil capsules had a significantly decreased score on the HRSD compared to those taking the placebo. Once again, the fish oil, even at this high dose, was well tolerated with no adverse events reported [52].

Various doses of pure EPA have also been investigated in depression. In a 12-week, randomized, double-blind, placebo-controlled study, patients (n = 70) were given ethyl-EPA at doses of 1 g, 2 g or 4 g. The patients in this case had experienced persistent depression, despite ongoing standard antidepressant pharmacotherapy at adequate does. Interestingly, in this study, "less was more." Those in the 1 g per day group had the best outcome. The patients who received 1 g per day of EPA were the only group to show statistically significant improvements. Among the 1 g/day group, 53 percent achieved a 50 percent reduction in HRSD scores. The 1 g EPA led to improvements in depression, anxiety, sleep, lassitude, libido, and suicidal ideation. These findings suggest that omega-3 fatty acids can augment antidepressant pharmacotherapy and/or alleviate depression by entirely different means than standard medications [53]. A large study examining the effects of omega-3 or placebo added to cognitive-behavior therapy would be of interest.

To date, the published data on supplementation with pure EPA on MDD or depressive symptoms have been positive. With regard to DHA or a combination of EPA and DHA, there have been three negative reports. A trial on DHA alone as monotherapy in the treatment of MDD was recently reported. In this study, 2 g pure DHA or placebo was administered to 36 patients with depression for six weeks. The response differences between the groups, as measured by scores on the Montgomery-Asberg Depression Rating Scale did not reach statistical significance [54]. In an open label pilot study, the combination of 1.7 g of EPA and 1.2 g of DHA failed to show benefits among seven women with a past history of post-partum depression. The omega-3 monotherapy was initiated between the 34^th ^– 36^th ^week of pregnancy and was assessed through 12 weeks post-partum. In these women the fish oil combination did not reduce the risk of relapse [55]. Finally, a pure DHA supplement, at low doses of 200 mg per day for 4 months post-partum, did not improve self-rated or diagnostic measures of depression over placebo. However, the women enrolled (n = 89) in this study were not clinically depressed as a group, which precludes interpretation that DHA is ineffective in post-partum depression [56].

## Other dietary considerations

It is important to consider the nutrients which can ultimately influence omega-3 status. Among them, four important dietary factors also relate to MDD: zinc, selenium, folic acid and dietary antioxidants. A number of studies have shown that zinc levels are lower among patients with depression and a recent study found that 25 mg zinc supplementation may improve depressive symptoms [57]. Interestingly, 25 mg of zinc supplemented for two months has also been shown to significantly increase omega-3 status in the plasma phospholipids at the expense of saturated fat [58]. Lowered levels of selenium have been associated with negative mood scores in at least 5 studies [59]. Selenium plays a significant role in the human antioxidant defense system. In addition, selenium deficiency can interfere with the normal conversion of ALA into EPA and DHA, and results in an increase in the omega-6:omega-3 ratio [60].

Regarding folic acid, a growing body of research has documented the low levels of folic acid among patients with depression [61]. In addition, there are small clinical trials showing a beneficial effect of folic acid in depression, and its ability to enhance the effectiveness of antidepressant medications at just 500 mcg [61,62]. It is of relevance here because folic acid has been shown to increase omega-3 status when supplemented, and decrease omega-3 status when it is in deficiency in the animal model [63]. In addition, a folic acid deficient diet can enhance lipid peroxidation [64].

In patients with MDD there are in fact signs of oxidative stress and lipid peroxidation, and antidepressant medications may reverse the severity of oxidative stress in depressed patients [65]. A recent human study found that depressive symptoms are independently correlated with lipid peroxidation [66]. Patients with obsessive compulsive disorder (OCD) and co-morbid depression have higher levels of lipid peroxidation than those with OCD alone [67]. Dietary antioxidants are known to influence the antioxidant defense system, and new research suggests that dietary antioxidants can influence omega-3 status. Specifically, a diet devoid of antioxidants lowered essential fatty acid levels in the plasma of trained athletes, even though the amount and types of fats were not altered [68]. Omega-3 fatty acids have been shown to decrease lipid peroxidation in vivo [69], and antioxidant supplementation can prevent the negative influence of saturated fat on BDNF levels and cognitive function in animals [70].

## Conclusion

While far from robust, there is enough epidemiological, laboratory and clinical evidence to suggest that omega-3 fatty acids may play a role in certain cases of depression. Fish oil supplements are well tolerated, and have been shown to be without significant side effects over large scale, 3-year research [71]. Generally, omega-3 supplements are inexpensive, which makes them an attractive option as an adjuvant to standard care. At this time, however, the routine use of omega-3 fatty acids for the treatment of MDD cannot be recommended.

The research reviewed here shows that the data is far from unequivocal. Large trials are warranted to truly determine efficacy, appropriate dosing and the potentially active components – EPA, DHA, or both. It is also clear that omega-3 intake occurs in dietary context, one that includes other important nutrients. Future research should consider the influence of zinc, selenium, folic acid and dietary antioxidant status to determine who may be a successful candidate for omega-3 supplementation.

In the meantime, given the current excess intake of omega-6 rich oils, and the emerging research on omega-3 fatty acids and MDD, all mental health professionals should at least ensure adequate intake of omega-3 fatty acids among patients with MDD. The current average North American intake of EPA and DHA is approximately 130 mg per day, well short of the minimum 650 mg recommended by the international panel of lipid experts [6]. While it is not necessary for mental health professionals to become clinical nutritionists, consideration of a patient's dietary quality may be worthwhile. Hopefully future research will determine if dietary modifications or supplementation can influence the outcome of standard care.
